# Altered circadian behavior and light sensing in mouse models of Alzheimer’s disease

**DOI:** 10.3389/fnagi.2023.1218193

**Published:** 2023-06-20

**Authors:** Thaddeus K. Weigel, Cherry L. Guo, Ali D. Güler, Heather A. Ferris

**Affiliations:** ^1^Department of Neuroscience, University of Virginia, Charlottesville, VA, United States; ^2^Department of Biology, University of Virginia, Charlottesville, VA, United States; ^3^Division of Endocrinology and Metabolism, University of Virginia, Charlottesville, VA, United States

**Keywords:** Alzheimer’s disease, circadian behavior, jet lag, masking, amyloid beta, microglia, retina

## Abstract

Circadian symptoms have long been observed in Alzheimer’s disease (AD) and often appear before cognitive symptoms, but the mechanisms underlying circadian alterations in AD are poorly understood. We studied circadian re-entrainment in AD model mice using a “jet lag” paradigm, observing their behavior on a running wheel after a 6 h advance in the light:dark cycle. Female 3xTg mice, which carry mutations producing progressive amyloid beta and tau pathology, re-entrained following jet lag more rapidly than age-matched wild type controls at both 8 and 13 months of age. This re-entrainment phenotype has not been previously reported in a murine AD model. Because microglia are activated in AD and in AD models, and inflammation can affect circadian rhythms, we hypothesized that microglia contribute to this re-entrainment phenotype. To test this, we used the colony stimulating factor 1 receptor (CSF1R) inhibitor PLX3397, which rapidly depletes microglia from the brain. Microglia depletion did not alter re-entrainment in either wild type or 3xTg mice, demonstrating that microglia activation is not acutely responsible for the re-entrainment phenotype. To test whether mutant tau pathology is necessary for this behavioral phenotype, we repeated the jet lag behavioral test with the 5xFAD mouse model, which develops amyloid plaques, but not neurofibrillary tangles. As with 3xTg mice, 7-month-old female 5xFAD mice re-entrained more rapidly than controls, demonstrating that mutant tau is not necessary for the re-entrainment phenotype. Because AD pathology affects the retina, we tested whether differences in light sensing may contribute to altered entrainment behavior. 3xTg mice demonstrated heightened negative masking, a circadian behavior measuring responses to different levels of light, and re-entrained dramatically faster than WT mice in a jet lag experiment performed in dim light. 3xTg mice show a heightened sensitivity to light as a circadian cue that may contribute to accelerated photic re-entrainment. Together, these experiments demonstrate novel circadian behavioral phenotypes with heightened responses to photic cues in AD model mice which are not dependent on tauopathy or microglia.

## Introduction

Altered circadian rhythms are a common symptom of Alzheimer’s disease (AD). These alterations appear early in the disease, before hallmarks such as memory impairment, amyloid-β (Aβ) plaques, and neurofibrillary tangles (Musiek et al., 2015). AD circadian symptoms include sleep disruptions and a greater severity of behavioral symptoms later in the day, known as sundowning. Circadian disruptions are also observed at the molecular level, with alterations in circadian clock gene expression in the brains of AD patients (Cermakian et al., 2011). These circadian alterations are particularly interesting because they may play a role in disease progression: sleep can facilitate Aβ clearance from the brain (Xie et al., 2013; Shokri-Kojori et al., 2018) and poor sleep quality in adulthood is a risk factor for AD later in life (Sabia et al., 2021). Additionally, sleep disruptions caused by altered circadian rhythms significantly increase the difficulty of caring for AD patients (Kang et al., 2009; Musiek et al., 2015). Thus understanding the mechanisms of circadian disruption in AD could have both important preventative and therapeutic potential.

Many circadian phenotypes seen in humans with AD are recapitulated in mouse models of AD. Certain AD models demonstrate changes to the free running period (the intrinsic period of an animal’s circadian behavior when kept in constant darkness) and activity in light or dark phases (Sheehan and Musiek, 2020). AD model mice also score better in anxiety tests earlier in their active period compared to later (Bedrosian et al., 2011), a phenotype reminiscent of sundowning in AD patients. Circadian alterations are recapitulated at the molecular level as well, with changes to the amplitude and phase of rhythmic clock gene expression in some AD models including 3xTg (Bellanti et al., 2017) and 5xFAD (Song et al., 2015) mice.

Other facets of circadian rhythms have been less well-studied in AD models. Entrainment is the process of synchronizing the biological circadian clock with the daily rhythm of the environment. In this study we tested circadian behavior in models of AD using a “jet lag” protocol. We found that female 3xTg mice re-entrain more rapidly than wild type (WT) controls. We then examined neuroinflammation, amyloid pathology, and changes to light sensing as possible contributors to this altered circadian behavior.

## Results

### 3xTg mice have accelerated circadian re-entrainment

**FIGURE 1:**
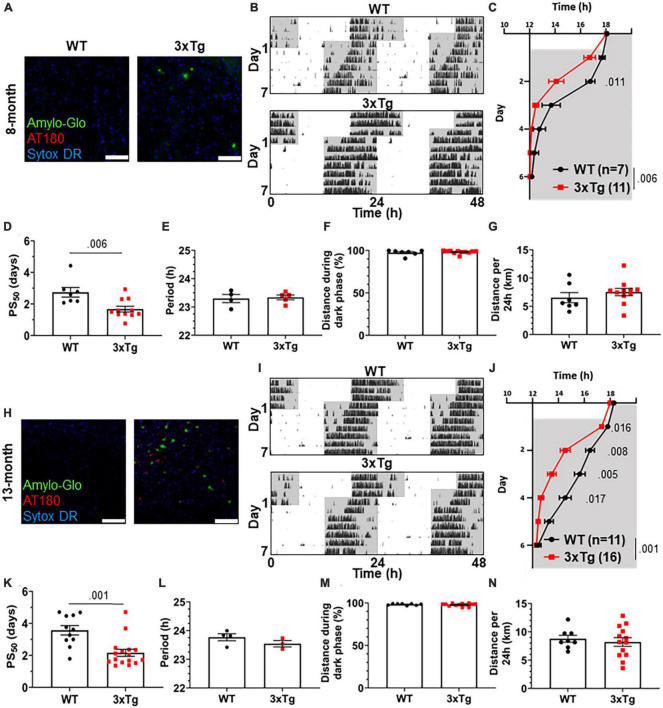
Altered circadian re-entrainment in 3xTg mice. **(A)** Representative images from the hippocampus of 8-month-old (mo) B6129SF2/J wild type (WT) and 3xTg mice. Aβ plaques are stained with Amylo-Glo (green), phosphorylated tau is stained with AT180 (red), and nuclei are stained with Sytox-DR. Scale bars = 100 μm. **(B)** Representative double-plotted actograms of 8 mo WT and 3xTg mice subjected to a 6 h phase advance. Light and dark phases of the LD cycle are represented by white and gray background, respectively. **(C)** Group analysis of activity onset in 8 mo mice, with gray representing darkness as in panel **(B)**. Mixed model with Sidak *post hoc* comparison, *n* = 7–11. **(D)** Time to 50% of total phase shift (PS_50_) in 8 mo mice from panel **(C)**, *n* = 7–11. **(E)** Free-running period (averaged over 7 days) in 8 mo mice maintained in constant darkness. **(F)** Percent of running performed during the dark phase and **(G)** total distance run in 24 h (averaged over two 24 h periods) in 8 mo mice, *n* = 7–11. **(H–N)** Same as panels **(B–G)** but in 13 mo mice. *n* = 11–16 in panels **(I–K)**, 3–4 in panel **(L)**, 8–13 in panels **(M,N)**. All analyses are two tailed Student’s *t*-tests unless otherwise noted. All data plotted as mean ± SEM.

To test the re-entrainment behavior of AD model mice, we first studied female 3xTg mice. The 3xTg mouse model of AD carries pathogenic mutations in amyloid precursor protein (APP), presenilin 1 (PS1), and human tau (MAPT), resulting in progressive accumulation in the brain of Aβ plaques and neurofibrillary tangles. Sex-specific circadian behavioral alterations have previously been observed in 3xTg mice (Sterniczuk et al., 2010), and female 3xTg mice have a more rapid progression of AD pathology than males (Dennison et al., 2021). In a photic phase shift experiment, which measures the shifting of circadian behavior caused by one pulse of light during the dark phase, female 3xTg mice showed a trend toward greater phase shifting while males did not (Sterniczuk et al., 2010). We examined re-entrainment in these mice using a shifted light-dark (LD) cycle, simulating travel across 6 time zones and subsequent “jet lag.” This behavior is not altered in male 3xTg mice (González-Luna et al., 2021), but female mice, which have more severe AD pathology than males, have not been studied in this paradigm. We allowed female 8-month-old (mo) 3xTg and B6129SF2/J wild type (WT) control mice to entrain to a 12:12 L:D light cycle and monitored their behavior on a running wheel. At this age, female 3xTg mice have only mild Aβ and tau pathology (Figure 1A). Plaques and phosphorylated tau are not observed in the SCN (Supplementary Figure 1A). After full entrainment and habituation to the running wheel, the LD cycle was advanced by 6 h (Figure 1B). The onset of nightly running was measured in the days following the light cycle shift. 3xTg activity onset was significantly earlier than WT following the light cycle shift on day 2 after the shift (Figure 1C), demonstrating more rapid re-entrainment. We calculated the number of days each mouse took to complete half of the re-entrainment, the 50% phase shift (PS_50_), and found that mean PS_50_ was 1.07 days earlier in 3xTg than WT mice (*p* < 0.006) (Figure 1D). We examined free-running period when kept in 24 h darkness (Figure 1E) and preference for running during the dark phase (Figure 1F) and found no difference between 3xTg and WT in these other aspects of circadian behavior. Total running was not affected by genotype (Figure 1G), suggesting that the wheel running re-entrainment phenotype is not influenced by the hyperactivity or perseverative behavior sometimes observed in AD mouse models.

To test this phenotype as AD pathology progresses with aging, we repeated this experiment using 13 mo female 3xTg and WT mice, which have more advanced amyloid and tau pathology (Figures 1H, I), though they still lack plaques and phosphorylated tau in the SCN (Supplementary Figure 1B). Behavior onset after the light cycle shift was significantly earlier in 13-month 3xTg than WT mice on days 1–4 after the shift (Figure 1J). Mean PS_50_ at 13 months was 1.41 days faster (*p* < 0.001) in 3xTg than WT (Figure 1K). Free running period and preference for running in the dark phase were not affected by genotype (Figures 1L, M). Total running was again not affected by genotype (Figure 1N). These results show that 3xTg mice re-entrain more rapidly in a jet lag paradigm at multiple stages of pathological progression.

3xTg mice exhibit a metabolic phenotype resulting in greater body weight than B6129SF2/J controls (Robison et al., 2020), and lost more weight during their time with running wheel access, but neither greater body weight nor greater weight loss correlated with more rapid re-entrainment (Supplementary Figures 2A–F). These metabolic differences are thus not likely to contribute to the observed re-entrainment phenotype.

### Microglia depletion does not alter circadian re-entrainment in 3xTg mice

**FIGURE 2:**
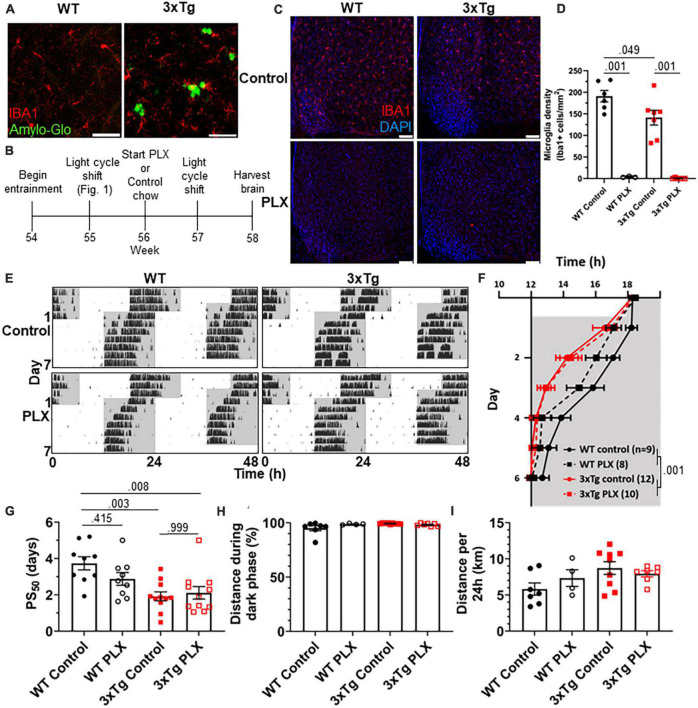
Microglia depletion does not rescue circadian re-entrainment phenotype in 3xTg mice. **(A)** Representative images of hippocampal microglia morphology in 13-month-old (mo) 3xTg and B6129SF2/J WT mice. Aβ plaques are stained with Amylo-Glo (green) and microglia are marked by staining for IBA1 (red). Scale bars = 50 μm. **(B)** Timeline of experiment. After completing the light cycle shift experiment in Figures 1H–N, 13 mo WT and 3xTg mice were fed Plexxikon 3397 (PLX) chow to deplete microglia or control chow for 7 days before beginning light cycle shift. **(C)** Representative images of microglia depletion in the SCN and surrounding region in WT and 3xTg mice fed control or PLX diets. Microglia labeled by staining for IBA1 (red). Scale bars = 100 μm. **(D)** Quantification of microglia in the ventromedial hypothalamus, the region containing the SCN, in WT and 3xTg mice fed control or PLX diets. **(E)** Representative double-plotted actograms of 13 mo wild type (WT) and 3xTg mice, treated with control or PLX chow, subjected to a 6 h phase advance. Light and dark phases of the LD cycle are represented by white and gray background, respectively. **(F)** Group analysis of activity onset, with gray representing darkness as in panel **(E)**, *n* = 8–12. Mixed model with Sidak *post hoc* comparison. **(G)** Time to 50% of total phase shift (PS_50_) in mice from panel **(E)**, *n* = 8–12. **(H)** Percent of running performed during the dark phase and **(I)** total distance run in 24 h (averaged over two 24 h periods), *n* = 4–9. All analyses are 2-way ANOVAs with Sidak *post hoc* comparison unless otherwise noted. All data plotted as mean ± SEM.

As neuronal loss does not appear in AD until long after the development of circadian symptoms, we sought other possible mechanisms underlying this behavioral phenotype. Microglia are heavily implicated in AD pathophysiology and microglia activation can contribute to disease progression. In the 3xTg model, the brain has elevated levels of microglia-produced pro-inflammatory cytokines (Park et al., 2021) and microglia activation and proliferation can be observed before the development of Aβ plaques (Janelsins et al., 2005). We observe activated microglia in 3xTg mice at 13 mo, where they cluster around Aβ plaques and display a more amoeboid morphology (Figure 2A). Microglia depletion in AD models decreases neuroinflammatory signaling without acutely altering amyloid and tau pathology and in some studies can partially restore memory deficits (Spangenberg et al., 2016). We hypothesized that activated microglia and neuroinflammation could contribute to the circadian re-entrainment phenotype observed in 3xTg mice and microglia depletion would rescue the re-entrainment phenotype. We used the colony stimulating factor 1 receptor (CSF1R) antagonist Plexxikon 3397 (PLX) to rapidly deplete microglia from the brain. Following re-entrainment to the shifted light cycle in Figure 1, 13-month 3xTg or WT mice were switched to control or PLX chow (600 mg/kg) for 7 days to deplete microglia (Figure 2B). PLX treatment effectively depleted microglia from the brain in WT and 3xTg mice, reducing the number of microglia in the ventromedial hypothalamus, the region containing the SCN, by > 98% (Figures 2C, D). After 7 days of PLX or control treatment, light cycles were advanced by 6 h and running wheel behavior monitored (Figure 2E). PLX treatment did not rescue the more rapid re-entrainment in 3xTg mice, and there was no significant effect of treatment on time of running onset (Figure 2F). As in Figure 1, behavior onsets were earlier in 3xTg than WT mice, with a significant effect of genotype on time of running onset (*p* < 0.001). PLX treatment did not significantly alter PS_50_ in 3xTg mice (Figure 2G). There was a non-significant trend toward higher PS_50_ in WT control vs. PLX-treated mice (*p* < 0.415). There was no significant effect of treatment on PS_50_ (*p* < 0.316), but the effect of genotype on PS_50_ was again significant (*p* < 0.001). Thus, acute microglia depletion did not rescue the re-entrainment phenotype observed in 3xTg mice. PLX treatment also did not alter other running behaviors measured, with no significant effect of genotype or treatment found in percent running during the dark phase or total distance traveled (Figures 2H, I). These data demonstrate that re-entrainment remains altered in microglia-depleted 3xTg mice, suggesting that microglia and neuroinflammation are not acutely responsible for this circadian phenotype.

### 5xFAD mice have accelerated circadian re-entrainment

**FIGURE 3:**
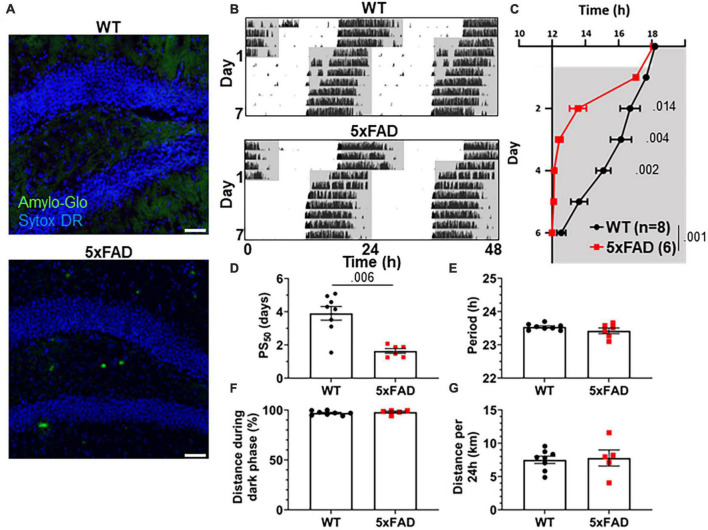
Altered circadian re-entrainment in 5xFAD mice. **(A)** Representative images from the hippocampus of 7-month-old (mo) 5xFAD and littermate control WT mice. Aβ plaques are stained with Amylo-Glo (green) and nuclei are stained with Sytox-DR (blue). Scale bars = 500 μm. **(B)** Representative double-plotted actograms of 7 mo WT and 5xFAD mice subjected to a 6 h phase advance. Light and dark phases of the LD cycle are represented by white and gray background, respectively. **(C)** Group analysis of activity onset, with gray representing darkness as in panel **(B)**. Mixed model with Sidak *post hoc* comparison, *n* = 6–8. **(D)** Time to 50% of total phase shift (PS_50_) in mice from panel **(C)**, *n* = 6–8. **(E)** Free-running period (averaged over 7 days) in mice maintained in constant darkness, *n* = 6–8. **(F)** Percent of running performed during the dark phase and **(G)** total distance run in 24 h (averaged over two 24 h periods), *n* = 6–8. All analyses are two tailed Student’s *t*-tests unless otherwise noted. All data plotted as mean ± SEM.

3xTg mice carry mutations driving pathological Aβ and tau expression in the brain. To probe whether both of these pathological proteins are necessary in order to produce the re-entrainment phenotype we observed in 3xTg mice, we studied re-entrainment in 5xFAD mice. The 5xFAD model expresses mutant APP and PS1 transgenes, but no mutant tau transgene, and thus develops aggressive amyloid pathology without neurofibrillary tangles. 5xFAD mice show altered molecular circadian rhythms and circadian behavior (Song et al., 2015; Lee et al., 2020). Based on the observed behavioral phenotype in 8 mo 3xTg mice, which have amyloid pathology but little tauopathy, we hypothesized that Aβ is sufficient to alter re-entrainment. We studied female 5xFAD mice at 7 months of age, at which time they have extensive Aβ plaques (Figure 3A), though none are detected in the SCN (Supplementary Figure 1C). We repeated the jet lag experiment described above in these aged 5xFAD mice (Figure 3B). Behavior onset was significantly earlier in 5xFAD mice than WT mice on days 2–4 after the shift (Figure 3C), and mean PS_50_ was reached 2.27 days earlier (*p* < 0.006) (Figure 3D). Free-running period and preference for running during the dark phase were not significantly affected by genotype (Figures 3E, F). Total running distance was not altered in 5xFAD mice, suggesting that hyperactivity or perseverative behavior were not responsible for altered performance on the running wheel in this model (Figure 3G). These results closely recapitulate the findings in aged 3xTg mice. Thus, amyloid pathology in the absence of mutant tau is sufficient to alter circadian re-entrainment in these AD mouse models.

5xFAD mice do not display the same increased bodyweight phenotype as 3xTg mice, and we again found no consistent correlation between body weight or weight loss during the running wheel period and speed of re-entrainment (Supplementary Figures 2G–I).

To test whether abnormal tau can drive this re-entrainment phenotype in the absence of Aβ pathology, we repeated the jet lag experiment with the PS19 mouse model, which carries a mutation driving aggressive tauopathy, but does not carry amyloidogenic mutations (Yoshiyama et al., 2007). Unlike the 3xTg and 5xFAD models, re-entrainment was not altered in female PS19 mice at 7 months (Supplementary Figure 3). The rapid re-entrainment of WT controls for this model may obscure fine differences between groups, but the absence of a trend toward more rapid re-entrainment in this tauopathy model further supports the hypothesis that the re-entrainment phenotype is driven by amyloid, rather than tau, pathology.

### AD model mice exhibit heightened sensitivity to photic cues

Light entering via the retina entrains the SCN circadian clock. Amyloid and tau pathology are detectable in the retina in AD (Hart et al., 2016; Koronyo et al., 2017), and the cells which provide photic inputs from the retina to the circadian system, the intrinsically photosensitive retinal ganglion cells (ipRGCs), are decreased in AD (La Morgia et al., 2016). We next examined whether altered re-entrainment in 3xTg mice could be influenced by altered light sensing in the retina, rather than altered circadian timekeeping in the SCN.

**FIGURE 4:**
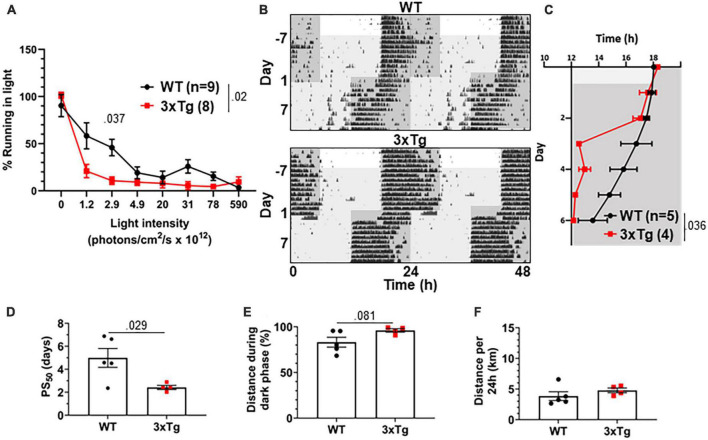
Visual circadian function is altered in 3xTg mice. **(A)** Reduction in running caused by light pulses of increasing intensity during the dark phase in 8-month-old 3xTg and B6129SF2/J WT mice, *n* = 8–9. Lights of different intensities were turned on from ZT13-14 and distance run in that period was compared to running during the same period of constant darkness the previous night. **(B)** Dim light jet lag trial representative double-plotted actograms. Lighting was switched from 590 × 10^12^ to 2.9 × 10^12^ photons/cm^2^/s. Seven days later, LD phase was advanced by 6 h. Dark phase represented by dark gray background, dim light represented by light gray. **(C)** Group analysis of activity onset, with gray representing darkness as in panel **(F)**. Mixed model with Sidak *post hoc* comparison, *n* = 4–5. **(D)** Time to 50% of total phase shift (PS_50_) in mice from panel **(C)**, *n* = 4–5. **(E)** Percent of running performed during the dark phase and **(F)** total distance run in 24 h (averaged over two 24 h periods), *n* = 4–5. All analyses are two tailed Student’s *t*-tests unless otherwise noted. All data plotted as mean ± SEM.

The jet lag re-entrainment paradigm relies on the retina to detect photic entrainment cues and the SCN to shift the biological clock in response to those cues. We next tested negative masking, a test of behavioral response to light that depends on the retina and is preserved in SCN-ablated animals (Redlin and Mrosovsky, 1999). Masking is a phenomenon wherein changes in light conditions can alter normally circadian-controlled behaviors without first altering the circadian pacemaker. For example, a mouse running in the dark may stop running if the lights are suddenly turned on, in spite of it still being the animal’s active phase. To measure negative masking, we gave a 1-h pulse of light of different intensities beginning 1 h after the onset of the dark phase of a 12:12 LD cycle and monitored running wheel activity of 8 mo 3xTg and WT mice during that time. By comparing to running during uninterrupted darkness, we could calculate the degree to which different intensities of light masked running behavior, and therefore measure the sensitivity of the mouse to circadian light cues in a non-SCN-dependent system. We found a significant difference in negative masking behavior between 3xTg and WT mice (*p* < 0.02), and 3xTg running was significantly more suppressed by low-intensity 2.9 × 10^12^ photons/cm^2^/s lighting than WT (*p* < 0.037) (Figure 4A). Unexpectedly, this demonstrates an elevated responsiveness to photic circadian signals in AD model mice, raising the possibility that increased sensitivity to the photic re-entrainment cue in the jet lag paradigm may contribute to more rapid re-entrainment.

To address this question, we repeated the jet lag experiment with these mice under dim lights. Mice were switched from the 590 × 10^12^ photons/cm^2^/s lighting conditions used for previous experiments to only 2.9 × 10^12^ photons/cm^2^/s, the intensity in the masking experiment where we found the most significant difference between 3xTg and WT. Mice were kept on a 12:12 LD cycle with these dim lights for 7 days, during which time all mice maintained their entrainment (Figure 4B). After 7 days the jet lag phase advancement was performed, still under dim lights, and re-entrainment was observed. 3xTg mice re-entrained significantly more rapidly than WT (*p* < 0.036) (Figure 4C), with a PS_50_ a dramatic 2.54 days earlier (*p* < 0.029) (Figure 4D). Also notably, after re-entrainment under dim light, some WT animals displayed unusual running behavior including considerable bouts of running before the onset of the dark phase (see Figure 4B, upper panel). This was not observed in 3xTg mice. This resulted in a trend (*p* = 0.081) toward decreased preference for running in the dark phase in WT mice (Figure 4E). Total running was not significantly different between the groups (Figure 4F). Overall, the masking and dim light experiments demonstrated that 3xTg mice respond more intensely than WT mice to dim light, both as a masking stimulus and an entrainment cue. This supports the hypothesis that altered retinal function contributes to differing circadian behavior.

**FIGURE 5:**
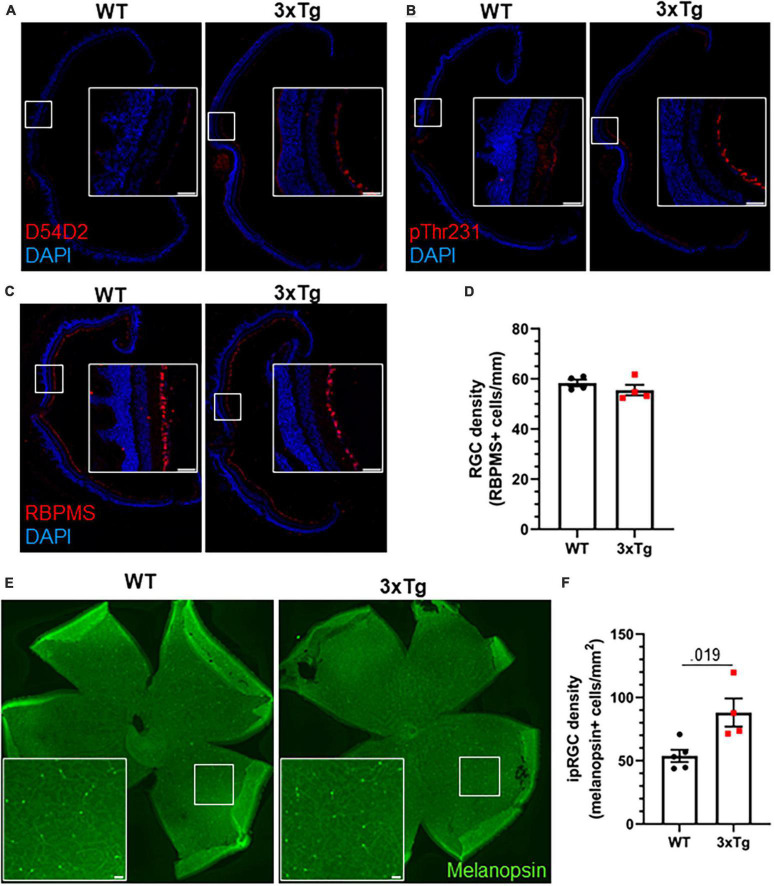
Increased ipRGC density in 3xTg retinas. Thirteen month old WT and 3xTg sagitally sectioned retinas were stained with DAPI and **(A)** D54D2 for Aβ, **(B)** pThr231 for phospho-tau, and **(C)** RBPMS for retinal ganglion cells. **(D)** Quantification of panel **(C)**. **(E)** Retina whole mounts from WT and 3xTg mice stained for melanopsin to identify ipRGCs. **(F)** Quantification of panel **(E)**. All analyses are two tailed Student’s *t*-tests. All data plotted as mean ± SEM. Scale bars = 50 μm.

We next analyzed the retinas of aged 3xTg mice by immunohistochemistry. Though we detected Aβ and tau phosphorylation in the retinas of 13 mo 3xTg mice (Figures 5A, B), we did not observe gross changes to retinal morphology or the RGC population (Figure 5C), and there was no significant decrease in RGC density in 3xTg retinas (*p* > 0.298) (Figure 5D). Interestingly, we found a 64% increase in the density of melanopsin-positive ipRGCs in the retinas of these 3xTg mice compared with WT (*p* < 0.019) (Figures 5E, F). All retinal tissue samples were collected between zeitgeber time (ZT) 5–8 and ipRGC density was averaged across images from multiple regions of the retina to minimize the effects of rhythmicity (Hannibal et al., 2005) and regional disparities (Dacey et al., 2005) in melanopsin expression. This increase in the ipRGC population may contribute to the heightened sensitivity to light as a circadian cue in 3xTg mice.

## Discussion

We found that two amyloid-based AD mouse models have significantly accelerated re-entrainment in a jet lag paradigm. This behavioral phenotype was not affected by the depletion of microglia and did not depend on the presence of tauopathy. These mice were also more sensitive to light in a trial of negative masking, an SCN-independent behavior, and had increased density of ipRGCs. Together these results uncover a novel circadian phenotype in AD model mice driven by Aβ.

This is the first report, to our knowledge, of altered jet lag behavior in AD model mice. Previous studies in APP/PS1 mice (Otalora et al., 2012; Kent et al., 2019) and male 3xTg mice (González-Luna et al., 2021) did not show altered re-entrainment in the jet lag paradigm. However, the APP/PS1 model has been criticized as not replicating some circadian phenotypes observed in AD patients (Sheehan and Musiek, 2020) and most studies find less severe pathophysiology and behavioral phenotypes in male than female 3xTg mice (Dennison et al., 2021). Given the variability between different AD models, our finding of the same robust re-entrainment phenotype in two different models is important for validating that this phenotype is not the result of peculiarities of a specific genetic model. We do not observe alterations to free running period or daily activity patterns, two measures of circadian behavior in which differences have been reported in some studies with 3xTg mice (Wu et al., 2018; Adler et al., 2019). Previous research has not found altered free running period in 5xFAD mice (Nagare et al., 2020), consistent with our findings here. We did observe notable differences between WT mice in our studies depending on age (Figures 1D, K) and strain (Figures 1D, 3D and Supplementary Figure 3C). Indeed, given the very rapid re-entrainment of littermate controls of PS19 mice, the B6C3 background strain in this model may not be well-suited to detecting fine differences in re-entrainment behavior, and other tauopathy models may exhibit behavioral differences. These heterogeneous results highlight the importance of sex, age, and background strain in circadian behavioral experiments.

Modulating neuroinflammation in AD is a promising field of research, and microglia can both contribute to and protect against disease progression. As neuroimmune activation and circadian disruptions both appear early in the progression of AD, and inflammation can modulate circadian rhythms, microglia seemed a promising possible mechanism underlying circadian symptoms. However, we found that microglia depletion did not rescue jet lag behavior in 3xTg mice. The microglia depletion in our experiment was acute and longer treatment may have other effects on circadian behavior, but our results suggest that acutely targeting inflammation in AD may not directly ameliorate circadian disruptions. Retinal microglia are also likely not acutely involved in the jet lag phenotype as CSF1R inhibitors effectively deplete microglia in the retina as well as in other regions of the brain (Dharmarajan et al., 2017; Huang et al., 2018). We did observe a trend toward accelerated re-entrainment in WT mice after microglia depletion, which may suggest a role for microglia in circadian regulation in the healthy brain. A study reported altered circadian behavior in rats after using a targeted diphtheria toxin approach to deplete microglia (Sominsky et al., 2021), although these results may have been influenced by sickness behavior induced by the diphtheria toxin approach. Another recent study found that microglia depletion alters sleep in mice (Liu et al., 2021), but did not study circadian-regulated behaviors. More research is needed to understand the effects of microglia on circadian rhythms in health and disease.

Accelerated recovery from jet lag is not one of the circadian symptoms of AD, but this phenotype may reflect underlying circadian disruptions. Strategies for managing sundowning and improving sleep-related symptoms in AD patients include maintaining strict light schedules, increasing exposure to intense light during daylight hours, and decreasing exposure to light during the night (Mitolo et al., 2018). AD patients also have decreased amplitude of rhythmic circadian gene expression (Cermakian et al., 2011). This may indicate a weak biological circadian clock, making them heavily dependent on external cues for its maintenance. Rapid re-entrainment may indicate a decrease in the coupling and synchrony of neurons in the SCN, resulting in an intrinsic circadian timekeeper less resistant to being shifted by misaligned photic entrainment cues. A very similar jet lag phenotype is observed in mice genetically knocked out for vasopressin signaling (Yamaguchi et al., 2013), which impairs interneuronal communication in the SCN. Some reports find decreased AVP expression in the SCN of AD patients (Liu et al., 2000; Harper et al., 2008), though this has been disputed (Wang et al., 2015), and previous reports have found decreased expression of AVP in the SCN of 3xTg mice (Sterniczuk et al., 2010). Further studies will be needed to determine if changes in SCN signaling also contribute to this phenotype and how amyloid pathology drives those changes.

Our finding of increased sensitivity to low-intensity photic circadian cues in an AD model suggests mechanisms outside the SCN may play a role in the jet lag phenotype. A previous study found increased negative masking in male 3xTg mice (González-Luna et al., 2021) but did not examine behavior at low light levels. The retinal degeneration in AD models, including 3xTg mice (Grimaldi et al., 2018), and ipRGC loss in AD (La Morgia et al., 2016) suggest decreased sensitivity, not increased. The limited research on visual ability in 3xTg mice has not reported deficits (King et al., 2018), while some decrease in RGC activity can be observed by electroretinography (Frame et al., 2022). Recent research found a loss of ipRGCs and decreased ipRGC projections to the SCN in the APP/PS1 model (Carrero et al., 2023). Our finding in 3xTg mice of heightened responses to photic cues in two circadian behavioral paradigms, as well as increased ipRGC density, runs contrary to these findings and suggests that in some models, or at some stages of disease progression, ipRGC signaling may be increased rather than decreased. Indeed, while human data show a decrease in ipRGCs in postmortem AD retinas (La Morgia et al., 2016), another study did not find deficits in the pupillary light reflex, an ipRGC-dependent task, in pre-symptomatic AD subjects (Oh et al., 2019). Thus the reported decline in ipRGC number in AD may be specific to disease stage. More research will be necessary to determine the causes of differences between disease stages. Some evidence suggests that ipRGC number declines with age in mice (Semo et al., 2003) and humans (La Morgia et al., 2010), which may slow re-entrainment in aged WT mice. ipRGC density in 3xTg retinas over aging should be studied to determine whether this decline is modified and whether that may contribute to the observed increased ipRGC count at 13 months. The heterogeneity of the ipRGC population should also be further studied in these mice. Some ipRGC subtypes express melanopsin at very low levels and would be undetectable by the IHC technique used here. One possible explanation for the increase in melanopsin + cells in the 3xTg retinas could be an increase in melanopsin expression in non-M1 ipRGCs, or a skew toward more M1 and M2 ipRGCs, rather than an increase in the number of the ipRGC population. As these non-M1 ipRGCs have different functions and projection patterns than M1 ipRGCs [reviewed in Do (2019)], such a change in the ipRGC population could have consequences for diverse visual circuits and behaviors.

In summary, we demonstrate that AD model mice exhibit strikingly altered circadian behavior, suggesting a heightened sensitivity to photic circadian cues. This appears to be driven by pathogenic Aβ and does not require the presence of microglia or mutant tau.

## Materials and methods

### Mice

All animal experiments were conducted in accordance with the University of Virginia Institutional Animal Care and Use Committee. Animals were housed in a temperature and humidity controlled vivarium (22–24°C, ∼40% humidity) and were provided with food and water *ad libitum*. 3xTg experiments were conducted with 8–13 mo female 3xTg mice on a B6129 background (Oddo et al., 2003) (Jackson Laboratory #034830), with age-matched B6129SF2/J (Jackson Laboratory #101045) females as wild type controls. 5xFAD experiments were conducted with 7 mo female heterozygous 5xFAD mice on a C57BL/6J background (Oakley et al., 2006) (Jackson Laboratory #034848), with littermates genotyped as not expressing the mutant transgene serving as wild type controls. PS19 experiments were conducted with 7 mo female heterozygous PS19 mice on a B6C3 background (Yoshiyama et al., 2007) (Jackson Laboratory #008169), with littermates genotyped as not expressing the mutant transgene serving as wild type controls. For microglia depletion experiments, mice were given chow formulated with PLX3397 (660 mg/kg) or control chow for 7 days before light cycle shift and were maintained on PLX or control chow for the remainder of the experiment.

### Behavioral analysis

Behavioral testing protocol was adapted from Grippo et al. (2017). Mice were individually housed in cages (Nalgene) containing running wheels in light-tight boxes which were illuminated with timed fluorescent lights (590 × 10^12^ photons/cm^2^/s). Wheel running data were collected and analyzed with ClockLab software (Actimetrics). Activity onset was automatically detected by ClockLab software and when necessary, corrected by eye by an experimenter blinded to genotype and treatment group. Mice were allowed to habituate to running wheel cages and entrained to a 12 h:12 h LD cycle for at least 7 days before experiments began.

In jet lag re-entrainment trials, the onset of the dark phase was abruptly advanced by 6 h and running wheel activity was recorded for at least 7 days after light cycle shift. PS_50_ values were calculated using Prism software (GraphPad) by fitting a sigmoid dose-response curve to onset times in days 0–6 after light cycle shift (Kiessling et al., 2010). Total running distance and preference for running in the dark were measured after all mice had completely re-entrained after a phase shift and were averaged across 2 days. To determine free running period, after all mice had completely re-entrained they were switched to DD and period was calculated from the onset of activity across 7 days.

In masking trials, light intensity was decreased by wrapping fluorescent lights in neutral density filter films and measured with a spectrometer (Sekonic Spectrometer C-800). Illuminance was measured in lux, and photon flux, summed from 380 to 780 nm, was calculated using formulas in the supplementary materials in Lucas et al. (2014). A 1hr light pulse was delivered from ZT13-14. Percent running in light was calculated by comparing to the activity measured during the same period of constant darkness on the preceding day for each mouse. Individual trials were separated from each other by 3–4 days.

### Histological analysis and imaging

All mice used for histological analysis were sacrificed from ZT 5–9. Mice were anesthetized with a ketamine:xylazine solution and transcardially perfused with chilled phosphate buffered saline (PBS) followed by 4% paraformaldehyde (PFA). Brains were dissected and post-fixed in 4% PFA for 24 h at 4°C. Brains were cryoprotected by incubating > 24 h in 15% sucrose in PBS followed by 30% sucrose before being frozen and sectioned at 30 μm with a cryostat (Leica CM 1950). For stains using primary antibodies raised in mice, sections were blocked using Mouse on Mouse blocking solution (Vector Laboratories) for 2 h at room temperature. Otherwise, brain sections were incubated with blocking solution (5% bovine serum albumin, 2% horse serum, 1% Triton X-100 in PBS) for 2 h at room temperature. Primary antibodies were diluted in blocking solution and placed on sections overnight at 4°C. Sections were washed and incubated with secondary antibodies, diluted in blocking solution, for 2 h at room temperature. Sections were washed and if applicable were treated with DAPI (Sigma-Aldrich) (1:2000 in PBS for 15 min), Sytox Deep Red (Thermo Scientific) (1:2000 in PBS for 30 min), and/or AmyloGlo (Biosensis) per manufacturer protocol. Sections were washed and then mounted with ProLong Gold mounting media (Thermo Fisher).

Retinas were dissected and stained as in Gao et al. (2022) with minor modifications. For retinal sectioning, eye cups were dissected and post-fixed in 4% PFA for 30 min at room temperature. Eye cups were cryoprotected in 30% sucrose in PBS overnight at 4°C, frozen, and sectioned on the cryostat at 14 μm. Sections were again post-fixed with 2% PFA for 30 min at room temperature. Sections were then blocked, stained, and mounted as described for brain sections above. For retina whole-mounts, eye cups were dissected and post-fixed with 2% PFA for 1h on ice. They were then washed and blocked with blocking solution described above for 2 h at room temperature. They were incubated with primary antibody diluted in blocking solution overnight on a shaker at 4°C. They were then washed and incubated with secondary antibody diluted in blocking solution overnight on a shaker at 4°C. Finally the retinas were removed from the eye cup, cut to create four quadrants, and flat mounted with ProLong Gold mounting media.

Primary antibodies used were anti-phospho-tau AT180 (mouse, Invitrogen MN1040, 1:250), anti-phospho-tau pThr231 (rabbit, Invitrogen 701056, 1:500), anti-RBPMS (rabbit, Abcam ab152101, 1:500), anti-Iba1 (rabbit, Wako 019-19741, 1:300), anti-amyloid D54d2 (rabbit, Cell Signaling 8243, 1:200), and anti-melanopsin (Panda et al., 2002) (rabbit, 1:2500). Secondary antibodies used were donkey anti-rabbit AlexaFluor 594 (Invitrogen A21207), donkey anti-rabbit AlexaFluor 647 (Invitrogen A32795), donkey anti-mouse AlexaFluor 594 (Invitrogen A21203), and donkey anti-mouse AlexaFluor 647 (Invitrogen A11012).

Images were acquired with a Keyence BZ-X800 fluorescence microscope and images were stitched using BZ-X800 Analyzer software. Cell quantification was performed with ImageJ. For quantification of microglia, a 0.36 mm^2^ square was drawn in the ventromedial hypothalamus and all Iba1+ cells were counted. For quantification of RGCs in sectioned retinal tissue, 500–700 μm lines were drawn along the RGC layer beginning 300 μm to either side of the optic nerve exit point and all RBPMS+ cells were counted. For quantification of ipRGCs in retina whole mounts, four 0.25 mm^2^ squares were drawn in each retina at equal distances from the optic nerve exit point and melanopsin+ cells were counted in each, with cell density being calculated as an average of these counts.

### Statistical analysis

All data are presented as mean ± SEM. Statistical analyses were performed with Prism software (GraphPad). Time of onset of running after jet lag was analyzed by mixed model with Sidak *post hoc* comparisons. In PLX experiment, other data were analyzed by 2-way ANOVA with Sidak *post hoc* comparisons. All other data were analyzed by two tailed Student’s *t*-test. Differences between groups were determined to be statistically significant when *p* < 0.05.

## Data availability statement

The original contributions presented in this study are included in the article/Supplementary material, further inquiries can be directed to the corresponding author.

## Ethics statement

The animal study was reviewed and approved by University of Virginia School of Medicine.

## Author contributions

TW, AG, and HF designed the study. TW performed the experiments. TW and CG collected and analyzed data. TW wrote the manuscript with input from CG, AG, and HF. All authors discussed the results, revised the manuscript, and approved the final version of the manuscript.

## References

[B1] Adler P. Mayne J. Walker K. Ning Z. Figeys D. (2019). Therapeutic targeting of casein kinase 1δ/ε in an Alzheimer’s disease mouse model. *J. Proteome Res.* 18 3383–3393. 10.1021/ACS.JPROTEOME.9B00312/ASSET/IMAGES/LARGE/PR-2019-00312Q_0004.JPEG 31334659

[B2] Bedrosian T. A. Herring K. L. Weil Z. M. Nelson R. J. (2011). Altered temporal patterns of anxiety in aged and amyloid precursor protein (APP) transgenic mice. *Proc. Natl. Acad. Sci. U.S.A.* 108 11686–11691. 10.1073/PNAS.1103098108/SUPPL_FILE/PNAS.201103098SI.PDF 21709248 PMC3136261

[B3] Bellanti F. Iannelli G. Blonda M. Tamborra R. Villani R. Romano A. (2017). Alterations of clock gene RNA expression in brain regions of a triple transgenic model of Alzheimer’s disease. *J. Alzheimers Dis.* 59 615–631. 10.3233/JAD-160942 28671110 PMC5523844

[B4] Carrero L. Antequera D. Alcalde I. Megías D. Figueiro-Silva J. Merayo-Lloves J. (2023). Disturbed circadian rhythm and retinal degeneration in a mouse model of Alzheimer’s disease. *Acta Neuropathol. Commun.* 11:55. 10.1186/S40478-023-01529-6/FIGURES/9 PMC10067208 37004084

[B5] Cermakian N. Waddington Lamont E. Boudreau P. Boivin D. B. (2011). Circadian clock gene expression in brain regions of Alzheimer’s disease patients and control subjects. *J. Biol. Rhythms* 26 160–170. 10.1177/0748730410395732 21454296

[B6] Dacey D. M. Liao H. W. Peterson B. B. Robinson F. R. Smith V. C. Pokomy J. (2005). Melanopsin-expressing ganglion cells in primate retina signal colour and irradiance and project to the LGN. *Nature* 433 749–754. 10.1038/nature03387 15716953

[B7] Dennison J. L. Ricciardi N. R. Lohse I. Volmar C. H. Wahlestedt C. (2021). Sexual dimorphism in the 3xTg-AD mouse model and its impact on pre-clinical research. *J. Alzheimers Dis.* 80 41–52. 10.3233/JAD-201014 33459720 PMC8075398

[B8] Dharmarajan S. Fisk D. L. Sorenson C. M. Sheibani N. Belecky-Adams T. L. (2017). Microglia activation is essential for BMP7-mediated retinal reactive gliosis. *J. Neuroinflammation* 14:76. 10.1186/S12974-017-0855-0/FIGURES/3 PMC5382432 28381236

[B9] Do M. T. H. (2019). Melanopsin and the intrinsically photosensitive retinal ganglion cells: Biophysics to behavior. *Neuron* 104 205–226. 10.1016/J.NEURON.2019.07.016 31647894 PMC6944442

[B10] Frame G. Schuller A. Smith M. A. Crish S. D. Dengler-Crish C. M. (2022). Alterations in retinal signaling across age and sex in 3xTg Alzheimer’s disease mice. *J. Alzheimers Dis.* 88 471–492. 10.3233/JAD-220016 35599482 PMC9398084

[B11] Gao J. Griner E. M. Liu M. Moy J. Provencio I. Liu X. (2022). Differential effects of experimental glaucoma on intrinsically photosensitive retinal ganglion cells in mice. *J. Comp. Neurol.* 530 1494–1506. 10.1002/CNE.25293 34958682 PMC9010357

[B12] González-Luna I. A. Díaz-Cintra S. Juárez-Tapia C. Miranda-Anaya M. Aguilar-Vázquez A. Díaz-Muñoz M. (2021). Changes in 24 h rhythmicity of spontaneous locomotor activity in the triple transgenic mouse for Alzheimer’s disease (3xTg-AD) in a jet lag protocol: Correlations with retinal sensitivity. *J. Circadian Rhythms* 19:7. 10.5334/jcr.214 34163535 PMC8194968

[B13] Grimaldi A. Brighi C. Peruzzi G. Ragozzino D. Bonanni V. Limatola C. (2018). Inflammation, neurodegeneration and protein aggregation in the retina as ocular biomarkers for Alzheimer’s disease in the 3xTg-AD mouse model. *Cell Death Dis.* 9:685. 10.1038/s41419-018-0740-5 29880901 PMC5992214

[B14] Grippo R. M. Purohit A. M. Zhang Q. Zweifel L. S. Güler A. D. (2017). Direct midbrain dopamine input to the suprachiasmatic nucleus accelerates circadian entrainment. *Curr. Biol.* 27 2465–2475.e3. 10.1016/j.cub.2017.06.084 28781050 PMC5568465

[B15] Hannibal J. Georg B. Hindersson P. Fahrenkrug J. (2005). Light and darkness regulate melanopsin in the retinal ganglion cells of the albino Wistar rat. *J. Mol. Neurosci.* 27 147–155. 10.1385/JMN:27:2:147/METRICS 16186625

[B16] Harper D. G. Stopa E. G. Kuo-Leblanc V. McKee A. C. Asayama K. Volicer L. (2008). Dorsomedial SCN neuronal subpopulations subserve different functions in human dementia. *Brain* 131 1609–1617. 10.1093/BRAIN/AWN049 18372313 PMC3286014

[B17] Hart N. J. Koronyo Y. Black K. L. Koronyo-Hamaoui M. (2016). Ocular indicators of Alzheimer’s: Exploring disease in the retina. *Acta Neuropathol.* 132 767–787. 10.1007/S00401-016-1613-6 27645291 PMC5106496

[B18] Huang Y. Xu Z. Xiong S. Qin G. Sun F. Yang J. (2018). Dual extra-retinal origins of microglia in the model of retinal microglia repopulation. *Cell Discov.* 4:9. 10.1038/s41421-018-0011-8 29507754 PMC5827656

[B19] Janelsins M. C. Mastrangelo M. A. Oddo S. LaFerla F. M. Federoff H. J. Bowers W. J. (2005). Early correlation of microglial activation with enhanced tumor necrosis factor-alpha and monocyte chemoattractant protein-1 expression specifically within the entorhinal cortex of triple transgenic Alzheimer’s disease mice. *J. Neuroinflammation* 2:23. 10.1186/1742-2094-2-23/FIGURES/3 PMC1276812 16232318

[B20] Kang J.-E. Lim M. M. Bateman R. J. Lee J. J. Smyth L. P. Cirrito J. R. (2009). Amyloid-beta dynamics are regulated by orexin and the sleep-wake cycle. *Science* 326 1005–1007. 10.1126/science.1180962 19779148 PMC2789838

[B21] Kent B. A. Michalik M. Marchant E. G. Yau K. W. Feldman H. H. Mistlberger R. E. (2019). Delayed daily activity and reduced NREM slow-wave power in the APPswe/PS1dE9 mouse model of Alzheimer’s disease. *Neurobiol. Aging* 78 74–86. 10.1016/J.NEUROBIOLAGING.2019.01.010 30884411

[B22] Kiessling S. Eichele G. Oster H. (2010). Adrenal glucocorticoids have a key role in circadian resynchronization in a mouse model of jet lag. *J. Clin. Investig.* 120 2600–2609. 10.1172/JCI41192 20577050 PMC2898589

[B23] King J. L. Wong A. A. Brown R. E. (2018). Age-related changes in the spatial frequency threshold of male and female 3xTg-AD mice using optomotry. *J. Alzheimers Dis.* 62 591–596. 10.3233/JAD-170805 29480178

[B24] Koronyo Y. Biggs D. Barron E. Boyer D. S. Pearlman J. A. Au W. J. (2017). Retinal amyloid pathology and proof-of-concept imaging trial in Alzheimer’s disease. *JCI Insight* 2:e93621. 10.1172/JCI.INSIGHT.93621 28814675 PMC5621887

[B25] La Morgia C. Ross-Cisneros F. N. Koronyo Y. Hannibal J. Gallassi R. Cantalupo G. (2016). Melanopsin retinal ganglion cell loss in Alzheimer disease. *Ann. Neurol.* 79 90–109. 10.1002/ANA.24548 26505992 PMC4737313

[B26] La Morgia C. Ross-Cisneros F. N. Sadun A. A. Hannibal J. Munarini A. Mantovani V. (2010). Melanopsin retinal ganglion cells are resistant to neurodegeneration in mitochondrial optic neuropathies. *Brain* 133 2426–2438. 10.1093/BRAIN/AWQ155 20659957 PMC3139936

[B27] Lee J. Kim D. E. Griffin P. Sheehan P. W. Kim D. H. Musiek E. S. (2020). Inhibition of REV-ERBs stimulates microglial amyloid-beta clearance and reduces amyloid plaque deposition in the 5XFAD mouse model of Alzheimer’s disease. *Aging Cell* 19:e13078. 10.1111/ACEL.13078 31800167 PMC6996949

[B28] Liu H. Wang X. Chen L. Chen L. Tsirka S. E. Ge S. (2021). Microglia modulate stable wakefulness via the thalamic reticular nucleus in mice. *Nat. Commun.* 12:4646. 10.1038/s41467-021-24915-x 34330901 PMC8324895

[B29] Liu R. Y. Zhou J. N. Hoogendijk W. J. G. van Heerikhuize J. Kamphorst W. Unmehopa U. A. (2000). Decreased vasopressin gene expression in the biological clock of alzheimer disease patients with and without depression. *J. Neuropathol. Exp. Neurol.* 59 314–322. 10.1093/JNEN/59.4.314 10759187

[B30] Lucas R. J. Peirson S. N. Berson D. M. Brown T. M. Cooper H. M. Czeisler C. A. (2014). Measuring and using light in the melanopsin age. *Trends Neurosci.* 37:1. 10.1016/J.TINS.2013.10.004 24287308 PMC4699304

[B31] Mitolo M. Tonon C. la Morgia C. Testa C. Carelli V. Lodi R. (2018). Effects of light treatment on sleep, cognition, mood, and behavior in Alzheimer’s disease: A systematic review. *Dement. Geriatr. Cogn. Disord.* 46 371–384. 10.1159/000494921 30537760

[B32] Musiek E. S. Xiong D. D. Holtzman D. M. (2015). Sleep, circadian rhythms, and the pathogenesis of Alzheimer disease. *Exp. Mol. Med.* 47:e148. 10.1038/emm.2014.121 25766617 PMC4351409

[B33] Nagare R. Possidente B. Lagalwar S. Figueiro M. G. (2020). Robust light–dark patterns and reduced amyloid load in an Alzheimer’s disease transgenic mouse model. *Sci. Rep.* 10:11436. 10.1038/s41598-020-68199-5 32651420 PMC7351709

[B34] Oakley H. Cole S. L. Logan S. Maus E. Shao P. Craft J. (2006). Intraneuronal beta-amyloid aggregates, neurodegeneration, and neuron loss in transgenic mice with five familial Alzheimer’s disease mutations: Potential factors in amyloid plaque formation. *J. Neurosci.* 26 10129–10140. 10.1523/JNEUROSCI.1202-06.2006 17021169 PMC6674618

[B35] Oddo S. Caccamo A. Shepherd J. D. Murphy M. P. Golde T. E. Kayed R. (2003). Triple-transgenic model of Alzheimer’s disease with plaques and tangles: Intracellular Aβ and synaptic dysfunction. *Neuron* 39 409–421. 10.1016/S0896-6273(03)00434-3 12895417

[B36] Oh A. J. Amore G. Sultan W. Asanad S. Park J. C. Romagnoli M. (2019). Pupillometry evaluation of melanopsin retinal ganglion cell function and sleep-wake activity in pre-symptomatic Alzheimer’s disease. *PLoS One* 14:e0226197. 10.1371/JOURNAL.PONE.0226197 31821378 PMC6903762

[B37] Otalora B. B. Popovic N. Gambini J. Popovic M. Viña J. Bonet-Costa V. (2012). Circadian system functionality, hippocampal oxidative stress, and spatial memory in the APPswe/PS1dE9 transgenic model of Alzheimer disease: Effects of melatonin or ramelteon. *Chronobiol. Int.* 29 822–834. 10.3109/07420528.2012.699119 22823866

[B38] Panda S. Sato T. K. Castrucci A. M. Rollag M. D. DeGrip W. J. Hogenesch J. B. (2002). Melanopsin (Opn4) requirement for normal light-induced circadian phase shifting. *Science* 298 2213–2216. 10.1126/SCIENCE.1076848/SUPPL_FILE/PANDA.SOM.PDF 12481141

[B39] Park J. S. Kam T. I. Lee S. Park H. Oh Y. Kwon S. H. (2021). Blocking microglial activation of reactive astrocytes is neuroprotective in models of Alzheimer’s disease. *Acta Neuropathol. Commun.* 9:78. 10.1186/S40478-021-01180-Z/FIGURES/6 PMC8074239 33902708

[B40] Redlin U. Mrosovsky N. (1999). Masking by light in hamsters with SCN lesions. *J. Comp. Physiol. A* 184 439–448. 10.1007/S003590050343 10377978

[B41] Robison L. S. Gannon O. J. Thomas M. A. Salinero A. E. Abi-Ghanem C. Poitelon Y. (2020). Role of sex and high-fat diet in metabolic and hypothalamic disturbances in the 3xTg-AD mouse model of Alzheimer’s disease. *J. Neuroinflammation* 17:285. 10.1186/S12974-020-01956-5 32993686 PMC7526387

[B42] Sabia S. Fayosse A. Dumurgier J. van Hees V. T. Paquet C. Sommerlad A. (2021). Association of sleep duration in middle and old age with incidence of dementia. *Nat. Commun.* 12:2289. 10.1038/s41467-021-22354-2 33879784 PMC8058039

[B43] Semo M. Peirson S. Lupi D. Lucas R. J. Jeffery G. Foster R. G. (2003). Melanopsin retinal ganglion cells and the maintenance of circadian and pupillary responses to light in aged rodless/coneless (rd/rd cl) mice. *Eur. J. Neurosci.* 17 1793–1801. 10.1046/J.1460-9568.2003.02616.X 12752778

[B44] Sheehan P. W. Musiek E. S. (2020). Evaluating circadian dysfunction in mouse models of Alzheimer’s disease: Where do we stand? *Front. Neurosci.* 14:703. 10.3389/fnins.2020.00703 32733196 PMC7358444

[B45] Shokri-Kojori E. Wang G. J. Wiers C. E. Demiral S. B. Guo M. Kim S. W. (2018). β-Amyloid accumulation in the human brain after one night of sleep deprivation. *Proc. Natl. Acad. Sci. U.S.A.* 115 4483–4488. 10.1073/PNAS.1721694115/SUPPL_FILE/PNAS.1721694115.SD01.XLSX 29632177 PMC5924922

[B46] Sominsky L. Dangel T. Malik S. De Luca S. N. Singewald N. Spencer S. J. (2021). Microglial ablation in rats disrupts the circadian system. *FASEB J.* 35:e21195. 10.1096/fj.202001555RR 33200466

[B47] Song H. Moon M. Choe H. K. Han D. H. Jang C. Kim A. (2015). Aβ-induced degradation of BMAL1 and CBP leads to circadian rhythm disruption in Alzheimer’s disease. *Mol. Neurodegener.* 10:13. 10.1186/S13024-015-0007-X/FIGURES/7 PMC4404698 25888034

[B48] Spangenberg E. E. Lee R. J. Najafi A. R. Rice R. A. Elmore M. R. P. Blurton-Jones M. (2016). Eliminating microglia in Alzheimer’s mice prevents neuronal loss without modulating amyloid-β pathology. *Brain* 139 1265–1281. 10.1093/BRAIN/AWW016 26921617 PMC5006229

[B49] Sterniczuk R. Dyck R. H. Laferla F. M. Antle M. C. (2010). Characterization of the 3xTg-AD mouse model of Alzheimer’s disease: Part 1. Circadian changes. *Brain Res.* 1348 139–148. 10.1016/j.brainres.2010.05.013 20471965

[B50] Wang J. L. Lim A. S. Chiang W. Y. Hsieh W. H. Lo M. T. Schneider J. A. (2015). Suprachiasmatic neuron numbers and rest–activity circadian rhythms in older humans. *Ann. Neurol.* 78 317–322. 10.1002/ANA.24432 25921596 PMC4515161

[B51] Wu M. Zhou F. Cao X. Yang J. Bai Y. Yan X. (2018). Abnormal circadian locomotor rhythms and Per gene expression in six-month-old triple transgenic mice model of Alzheimer’s disease. *Neurosci. Lett.* 676 13–18. 10.1016/J.NEULET.2018.04.008 29626648

[B52] Xie L. Kang H. Xu Q. Chen M. J. Liao Y. Thiyagarajan M. (2013). Sleep drives metabolite clearance from the adult brain. *Science* 342 373–377. 10.1126/SCIENCE.1241224/SUPPL_FILE/XIE-SM.PDF 24136970 PMC3880190

[B53] Yamaguchi Y. Suzuki T. Mizoro Y. Kori H. Okada K. Chen Y. (2013). Mice genetically deficient in vasopressin V1a and V1b receptors are resistant to jet lag. *Science* 342 85–90. 10.1126/SCIENCE.1238599/SUPPL_FILE/1238599S3.MP4 24092737

[B54] Yoshiyama Y. Higuchi M. Zhang B. Huang S. M. Iwata N. Saido T. C. C. (2007). Synapse loss and microglial activation precede tangles in a P301S tauopathy mouse model. *Neuron* 53 337–351. 10.1016/J.NEURON.2007.01.010 17270732

